# Ferroptosis and recurrent miscarriage: a critical review of pathophysiology and emerging therapeutic targets

**DOI:** 10.3389/fcell.2025.1559300

**Published:** 2025-06-09

**Authors:** Mohammad Masoud Khodaei, Zahra Noori, Fateme Zare, Ahmad Meshkin

**Affiliations:** Reproductive Immunology Research Center, Shahid Sadoughi University of Medical Sciences, Yazd, Iran

**Keywords:** ferroptosis, recurrent miscarriage (RM), therapeutic interventions, trophoblast dysfunction, glutathione peroxidase 4

## Abstract

Ferroptosis is characterized as a specialized type of regulated cellular death that relies on iron and lipid peroxidation, which has recently been highlighted as playing a crucial role in the etiology of recurrent miscarriage (RM). Ferroptosis in RM is driven by dysregulated iron metabolism and increased oxidative stress, resulting from impaired antioxidant defense, which leads to lipid peroxidation and consequent cell death in trophoblasts. The cellular changes compromise placental development and impair trophoblast invasion and maternal-fetal tolerance. Therapeutic interventions targeting ferroptosis are promising for the mitigation of its effects and improvement of pregnancy outcomes. Strategies include Glutathione Peroxidase 4 (GPX4) activity enhancement, glutathione replenishment, ferroptosis inhibitors, and iron metabolism modulation. Further, new strategies targeting non-coding RNAs, and epigenetic regulators emphasize ferroptosis as a viable therapeutic target. This review emphasizes the importance of ferroptosis in the pathophysiology of RM and highlights its potential for guiding innovative treatments.

## 1 Introduction

The field of ferroptosis research has experienced significant expansion in recent years since the term was introduced in 2012. This distinct form of cell death, characterized by iron-dependent phospholipid peroxidation, is influenced by various cellular metabolic processes, including redox balance, iron metabolism, mitochondrial function, and the metabolism of amino acids, lipids, and carbohydrates, alongside numerous signaling pathways pertinent to various diseases (Jiang et al., 2021).

Recent studies indicate a correlation between ferroptosis and the emergence of female reproductive disorders, such as polycystic ovary syndrome, premature ovarian insufficiency, endometriosis, ovarian cancer, preeclampsia, and spontaneous abortion. The pathways and genes linked to ferroptosis may play a role in regulating granulosa cell growth and secretion, oocyte maturation, ovarian reserve capacity, early embryonic development, and placental oxidative stress (Liu M. et al., 2023).

Recurrent miscarriage is typically defined as the occurrence of three or more spontaneous abortions before 20–28 weeks of gestation. RM affects approximately 2%–5% of women of reproductive age, causing significant psychological distress and economic challenges. Contributing factors may include female age, anatomical and chromosomal irregularities, genetic predispositions, endocrinological issues, placental abnormalities, infections, lifestyle choices such as smoking and alcohol use, psychological influences, and exposure to environmental hazards like heavy metals, pollution, and radiation (La et al., 2021).

Despite ongoing research, the underlying causes of RM remain elusive, hindering effective treatment options and adding to the emotional burden on patients. It is recognized that a successful pregnancy requires a well-regulated immune response, suggesting that immunological imbalances may be a fundamental factor in adverse pregnancy outcomes (Li D. et al., 2021).

This study aims to elucidate the mechanisms through which ferroptosis affects trophoblast function and pregnancy outcomes, while also exploring potential therapeutic avenues to mitigate its adverse effects in cases of RM.

The terms Recurrent Miscarriage (RM), Recurrent Spontaneous Abortion (RSA), and Recurrent Pregnancy Loss (RPL) are often used interchangeably in the literature, although slight differences exist. RM and RSA typically refer to three or more consecutive pregnancy losses occurring before 20 weeks of gestation, with RSA often used in American contexts and RM more broadly. RPL is a more inclusive term that may cover pregnancy losses beyond 20 weeks, including biochemical pregnancies. For the purpose of this review, we use the term Recurrent Miscarriage (RM) consistently to reflect early pregnancy loss, aligning with clinical definitions and our research focus.

## 2 Methodology

We conducted a structured literature search using PubMed, Web of Science, and Scopus databases for studies published between January 2010 and March 2025. The keywords used were: “ferroptosis,” “recurrent miscarriage,” “recurrent pregnancy loss,” “decidua,” “trophoblast,” “oxidative stress,” and “iron metabolism.”

Inclusion criteria:

• Original research articles and reviews focusing on ferroptosis and recurrent miscarriage.
• English-language publications.
• Studies that evaluated ferroptosis-related genes, proteins, pathways, or biomarkers in the context of reproductive failure.

Exclusion criteria:

• Non-English articles.
• Conference abstracts, case reports, and editorials.
• Studies lacking relevance to both ferroptosis and miscarriage.

The final list of articles was curated based on title/abstract screening and full-text evaluation. In Table 1 we showed a summary of key studies investigating ferroptosis in recurrent miscarriage and related disorders (Table 1).

**TABLE 1:** Summary of key studies investigating ferroptosis in recurrent miscarriage and related disorders.

Author (Year)	Study type	Model/System	Objective	Biomarkers studied	Main findings
Sun et al. (2023)	Experimental	Human decidual stromal cells	To evaluate ferroptosis and iron metabolism in RM	GPX4, GSH, ROS, iron, ferroportin	Local iron overload induced ferroptosis; treatment with ferroptosis inhibitors improved pregnancy outcomes
Wang et al. (2023)	*In vitro*	Human trophoblast cells + LPS	To assess inflammation-induced ferroptosis	IL-6, IL-1β, TNF-α, GPX4, GSH, MDA, Fe^2+^, TCL6, miR-485-5p, TFRC	LPS exposure increased inflammation, ferroptosis, and apoptosis
Qin et al. (2024)	*In vitro*	Cytotrophoblast cells from RM patients	To explore epigenetic regulation of ferroptosis in RM	ALKBH5, FTL, N6-methyladenosine (m6A)	ALKBH5 attenuated ferroptosis by upregulating FTL expression via m6A RNA modification
Lai et al. (2024)	*In vivo*	CBA/DBA RM mouse model	To investigate the mechanism of Jianwei Shoutai Pill in RM	GPX4, SLC7A11, MDA, GSH, ACSL4, iron	Shoutai Pill reduced ferroptosis and improved fetal survival in RM mice
Zhang et al. (2023)	*In vivo* and *in vitro*	HUVECs, decidual tissues and serum from RM patients; placental tissues from mouse model	To assess environmental pollutant-induced ferroptosis	MARCHF1, GPX4, Fe^2+^, VEGFA, ANG-1	Benzo[a]pyrene (BaP) impaired angiogenesis and induced ferroptosis, contributing to miscarriage
Dai et al. (2024)	*In vitro*	Villous tissue of RM patients	To investigate IGF2BP3’s role in ferroptosis	IGF2BP3, GPX4	Downregulation of IGF2BP3 decreased GPX4 mRNA stability, promoting ferroptosis
Tian et al. (2024)	*In vivo* and *in vitro*	Hypoxic mouse model	To explore the lnc-HZ06/HIF1α-SUMO/NCOA4 regulatory axis	lnc-HZ06, HIF1α, NCOA4	Hypoxia induced ferroptosis in trophoblast cells via the HIF1α/NCOA4 pathway, leading to miscarriage
Li et al. (2024)	*In vitro* and animal model	Trophoblast cells	To examine the role of CRY2 in ferroptosis and trophoblast migration	CRY2, BMAL1, GPX4, MDA, iron ions, ferrostatin-1	CRY2 promoted ferroptosis and impaired migration; ferrostatin-1 reversed this dysfunction
Zeng et al. (2025)	Bioinformatics	Early missed abortion gene datasets	To identify ferroptosis-related genes in early miscarriage	TP53, EZH2, TIMP1, SLC3A2, GABARAPL2	Identified key ferroptosis-related genes potentially associated with early pregnancy loss

GPX4: Glutathione Peroxidase 4, GSH: glutathione, ROS: reactive oxygen species, IL-6: Interleukin-6, Interleukin-1 beta, TNF-α: Tumor Necrosis Factor-alpha, MDA: malondialdehyde, TCL6: T-cell Leukemia/Lymphoma 6,TFRC: transferrin receptor, ALKBH5: AlkB Homolog 5, RNA, demethylase, FTL: ferritin light chain, SLC7A11: Solute Carrier Family 7 Member 11, ACSL4: Acyl-CoA, Synthetase Long Chain Family Member 4, HUVECs: Human Umbilical Vein Endothelial Cells, BaP: Benzo[a]pyrene, MARCHF1: Membrane Associated Ring-CH-Type Finger 1, VEGFA: Vascular Endothelial Growth Factor A, ANG-1: Angiopoietin-1, IGF2BP3: Insulin-like Growth Factor 2 mRNA-binding Protein 3, lnc-HZ06: Long Non-coding RNA HZ06, HIF1α: Hypoxia-inducible Factor 1-alpha, NCOA4: Nuclear Receptor Coactivator 4, CRY2: Cryptochrome Circadian Regulator 2, BMAL1: Brain and Muscle ARNT-Like 1, TP53: Tumor Protein p53, EZH2: Enhancer of Zeste Homolog 2, TIMP1: Tissue Inhibitor of Metalloproteinases 1, SLC3A2: Solute Carrier Family 3 Member 2, GABARAPL2: GABA, Type A Receptor-Associated Protein Like 2, Fer-1: ferrostatin-1.

## 3 The ferroptosis pathway

### 3.1 Definition and distinctive features

Ferroptosis is a form of regulated cell death characterized by the accumulation of iron-dependent lipid peroxides, distinct from apoptosis, necroptosis, and autophagy. This process is morphologically marked by small mitochondria with condensed membrane densities, and biochemically by iron overload and GSH depletion, leading to loss of antioxidant defense and irreversible oxidative damage (Aguilera et al., 2021; Hasan et al., 2023).

### 3.2 Key molecular drivers of ferroptosis

Iron Overload: Excess intracellular ferrous iron (Fe^2+^) catalyzes the Fenton reaction, producing hydroxyl radicals (•OH) that initiate lipid peroxidation cascades. Disruption in iron homeostasis, including increased transferrin receptor expression and ferritin degradation via NCOA4-mediated ferritinophagy, contributes to ferroptotic susceptibility (Kuang et al., 2020).

Lipid Peroxidation: Polyunsaturated fatty acids (PUFAs), especially arachidonic and adrenic acids, are substrates for lipoxygenases (LOX), which drive peroxidation in membrane phospholipids, generating cytotoxic lipid peroxides such as 4-hydroxynonenal (4-HNE) and malondialdehyde (MDA) (Hasan et al., 2023).

Glutathione Depletion and GPX4 Inactivation: System Xc^−^ imports cystine for GSH synthesis. When this system is inhibited, GSH synthesis declines, leading to inactivation of GPX4. As GPX4 reduces phospholipid hydroperoxides, its dysfunction results in lethal lipid reactive oxygen species) ROS (accumulation (Pei et al., 2023).

Ferroptosis also interfaces with immune and inflammatory pathways. For instance, the release of damage-associated molecular patterns (DAMPs) such as HMGB1 during ferroptosis activates receptors like AGER/RAGE, stimulating the NF-κB pathway (Wen et al., 2019). These immune responses may contribute to tissue injury and pregnancy complications. Understanding these mechanisms helps link ferroptosis to disorders such as recurrent miscarriage, which will be explored in the next sections.

## 4 Ferroptosis in recurrent miscarriage

A variety of pathophysiological mechanisms contribute to RM, including genetic, anatomical, hormonal, and immunological factors. Immune dysregulation—particularly involving T-helper cell imbalance, increased pro-inflammatory cytokines (e.g., TNF-α, IL-6), and reduced regulatory T cell function—has been shown to impair maternal-fetal tolerance. Oxidative stress is another critical factor, with excess ROS damaging trophoblast cells and leading to placental insufficiency. Apoptosis, impaired trophoblast invasion, and abnormal angiogenesis have all been implicated in the etiology of RM.

Recently, ferroptosis, a distinct form of regulated cell death driven by iron overload and lipid peroxidation, has emerged as a novel mechanism potentially involved in RM. The following sections will explore the role of ferroptosis and its interaction with these established pathways.

During pregnancy, proper regulation of immune tolerance, oxidative stress, and cell death is critical for the maintenance of the decidua and placenta. Dysregulation of these processes, including ferroptosis, can contribute to pregnancy complications such as RM. ferroptosis and immunity are closely related to the normal physiological function of the endometrium and may play a role in the pathogenesis of RM and unexplained infertility.

The decidua, a specialized tissue of the endometrium, supports embryo implantation and immunomodulation. Aberrant decidual ferroptosis may result in defective implantation or impaired immune function. The decidua represents the maternal tissue most closely associated with the placental fetal unit. Notably, the endometrium/decidua is rich in immune cells, particularly uterine natural killer cells and macrophages, which originate from the bone marrow and selectively migrate to the uterine lining via the bloodstream. Decidualization involves functional and structural changes that occur within the endometrium, leading to the formation of the decidual lining that accommodates the implantation of the blastocyst. These changes include the recruitment of leukocytes and, importantly, the conversion of endometrial stromal fibroblasts into decidual stromal cells (DSCs). This reprogramming involves the downregulation of genes associated with proinflammatory responses and resistance to tissue invasion, along with the upregulation of genes that enhance cell proliferation, promote tolerance, and support tissue invasion (Sun et al., 2023). In a study, lipopolysaccharide (LPS) was used to induce inflammation in trophoblast cells during pregnancy. The inflammatory response was assessed by measuring proinflammatory cytokines such as interleukin (IL)-1β, IL-6, and TNF-α, and ferroptosis was assessed by analyzing MDA, GSH, GPX, and free iron (II) levels. Findings showed that LPS treatment resulted in decreased cell viability, invasion, and migration, while simultaneously increasing apoptosis, ferroptosis, and inflammation (Wang et al., 2023). IL-6 and TNF-α act through the JAK-STAT pathway. As a downstream target of this pathway, phosphorylated STAT3 can increase hepcidin expression to inhibit iron export and lead to ferroptosis. In other words, this evidence suggests that the inflammatory cytokines IL6 and TNF-α are associated with ferroptosis (Chen et al., 2023a). Regulatory T cells play an important role in mediating anti-inflammatory, immunosuppressive, and vasorelaxant activities essential for pregnancy establishment. They are remarkably able to limit and resolve inflammation and maintain tissue homeostasis through the secretion of cytokines such as IL-10 and transforming growth factor β (TGFβ) (Larsen et al., 2013). Macrophages display the capacity to polarize their functions along a spectrum between two distinct phenotypes (M1 and M2) in response to environmental cues. Factors such as LPS, IFN-γ, and TNF-α drive macrophages toward the M1 phenotype, resulting in the production of inflammatory cytokines including IL-1, IL-12, IL-18, and TNF-α. Conversely, M2 macrophage activation can be stimulated by cytokines such as IL-4, IL-13, IL-10, and TGFB1 (Yang et al., 2022). Increasing evidence suggests that macrophage polarization and ferroptosis can influence each other at the cell level independently or through interactions with other cells in a context-dependent manner. Therefore, it is likely that inflammatory cytokines play a role in pregnancy failure by increasing fetal ferroptosis, and conversely, anti-inflammatory cytokines act to reduce fetal ferroptosis and promote successful pregnancy.

As previously mentioned, one of the hallmarks of ferroptosis is iron accumulation. Iron plays an important role in many essential biological functions. However, excessive iron accumulation poses significant risks due to the reactive and toxic nature of sensitive iron. The simultaneous presence of both low serum iron levels and localized iron deposition in the decidua has been described in patients with RM. Mouse models of a low-iron diet exhibit similar iron deposition in the decidua, which also correlates with poor pregnancy outcomes. A likely contributor to this iron accumulation is the reduction in ferroportin expression that inhibits iron export from DSCs. This local iron overload in DSCs triggers ferroptosis through reduced GSH and glutathione peroxidase four levels with the concomitant increase in lipid and cellular ROS. Therapeutic interventions have shown promise in ameliorating these effects. Iron supplementation reduces iron accumulation in DSCs and improves pregnancy outcomes in models of spontaneous abortion. Moreover, supplementation with GSH and cystine, which are important components for GSH synthesis, significantly reduces ROS levels and inhibits DSC ferroptosis. Combining ferroptosis inhibitors with cysteine and GSH supplementation has been particularly effective in greatly reducing DSC ferroptosis and fetal loss in both the spontaneous and LPS-induced abortion models. These data hint at the therapeutic potential of targeting ferroptosis pathways in RM (Sun et al., 2023). Some studies have also shown that excess iron in follicular fluid increases the risk of endometriosis-associated infertility. In cases of endometriosis, iron overload has been shown to interfere with blastocyst formation, reduce GPX4 expression, and enhance lipid peroxidation in mouse models. These findings suggest that iron overload may lead to embryotoxic effects and induce ferroptosis (Li S. et al., 2021; Ni et al., 2022; Moghaddam et al., 2022).

Research shows that cigarette smoke negatively affects placental development, compromises fetal health, and reduces maternal fertility, leading to outcomes such as spontaneous abortion. In rat placentas, cigarette smoke has been observed to downregulate the expression of anti-ferroptosis regulators while upregulating pro-ferroptosis regulators. An *in vitro* study using HTR-8/SV neo (HTR-8) cells demonstrated that cigarette smoke exposure resulted in the accumulation of free iron and ROS, which subsequently caused lipid peroxidation and cell death. It was found that the use of inhibitors of ferroptosis could reduce cigarette smoke-induced cell death, suggesting that ferroptosis plays an important role in cigarette smoke-induced trophoblast cell death and may contribute to miscarriage (Guan et al., 2022).

## 5 Pathophysiological mechanism

### 5.1 Molecular mechanisms of ferroptosis

Ferroptosis is a regulated form of cell death characterized by iron-dependent lipid peroxidation and oxidative stress, playing a significant role in RM through trophoblast dysfunction. Iron dysregulation is a hallmark of ferroptosis, where excess intracellular iron promotes the Fenton reaction, generating hydroxyl radicals and amplifying oxidative stress. Dysregulated iron metabolism, including increased expression of transferrin receptors and ferritin degradation, leads to iron accumulation, contributing to ferroptosis (Chen et al., 2023b). The involvement of iron in inducing ferroptosis is largely mediated through two distinct pathways. First, iron can directly catalyze an overflow of lipid-derived ROS through the Fenton reaction. Second, Fe^2+^ acts as a cofactor for LOXs and prolyl hydroxylase, which are critical enzymes involved in lipid peroxidation and oxygen homeostasis (Conrad and Pratt, 2019; Doll and Conrad, 2017; Kagan et al., 2017).

During the first trimester, hepcidin levels increase, while in the second trimester, hepcidin levels decrease despite stable serum iron levels. In the case of spontaneous abortion, hepcidin, serum iron, and ferritin levels are high, showing that maternal iron metabolism is disturbed (Guo et al., 2019). Excessive iron has a significant impact on fertility outcomes, especially RM. Although RM patients often exhibit low serum iron levels, their DSCs locally accumulate iron, contributing to ferroptosis. This was hypothesized to be due to decreased ferroportin expression in DSCs, impairing iron export. Consequently, the overload of iron in these cells might lead to increased ROS production and mitochondrial dysfunction, triggering ferroptosis and potentially causing pregnancy loss. Results from mouse models suggest that these adverse effects could be mitigated by iron supplementation or inhibition of ferroptosis, thereby highlighting possible therapeutic approaches (Sun et al., 2023).

Lipid peroxidation is a key driver of ferroptosis, with PUFAs in cellular membranes particularly susceptible. Lipoxygenases enzymatically oxidize PUFAs, leading to lethal lipid peroxidation and cell death (Yang et al., 2016). GPX4 plays a critical role in preventing ferroptosis by reducing lipid hydroperoxides. Dysfunction of GPX4, often due to glutathione depletion, renders cells vulnerable to ferroptosis. The cystine/glutamate antiporter (System Xc^−^) is essential for glutathione synthesis, and its dysfunction exacerbates ferroptosis by impairing cystine uptake (Li et al., 2022).

**FIGURE 1:**
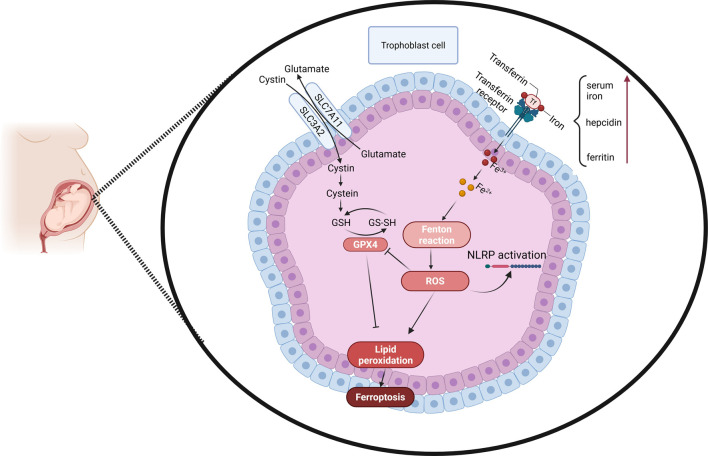
Molecular insights into ferroptosis and its role in the pathophysiology of recurrent pregnancy loss: Mechanisms of lipid peroxidation, oxidative stress, and cellular dysfunction in trophoblasts. Note: GPX4: Glutathione peroxidase 4, ROS: Reactive oxygen species, GSH: Glutathione, GS-SH: Glutathione disulfide, SLC7A11: Solute carrier family 7 member 11, SLC3A2: Solute carrier family 3 member 2, NLRP: Nod-like receptor protein.

ROS are central to ferroptosis, as they drive lipid peroxidation. During hypoxia-reperfusion cycles, ROS production increases, overwhelming the antioxidant defense system and promoting ferroptosis in trophoblast cells (Tian et al., 2024). The study conducted by Li Meihe demonstrates that trophoblast ferroptosis is a key contributor to miscarriage, which is regulated through oxidative stress and activation of the Nod-Like Receptor Protein (NLRP1) inflammasome. This type of stress disturbs the cellular balance by decreasing antioxidants like GSH and negatively affecting GPX4. Failure of these protective mechanisms increases transferrin receptor and acyl-CoA synthetase long-chain family member 4 (ACSL4) expression, enhancing lipid peroxidation and ferroptotic cell death. In parallel, oxidative stress triggers inflammatory signaling cascades involving caspase-1 activation and IL-1β production, leading to NLRP1 inflammasome activation in trophoblasts. This inflammasome-driven inflammation exacerbates cellular damage in a vicious cycle between ferroptosis and inflammation. This interaction interferes with the placental milieu, compromising the trophoblast’s role in nutrient exchange and leading to pregnancy complications such as miscarriage (Figure 1; Meihe et al., 2021).

### 5.2 Crosstalk with immune dysregulation

Ferroptosis, significantly impacts immune regulation, influencing the pathophysiology of RM through interactions with the maternal immune system. Ferroptotic cells release DAMPs, such as oxidized phospholipids, which activate innate immune responses. These molecules act as signals that recruit inflammatory cells, including neutrophils and macrophages, to the site of cell death, exacerbating local inflammation and potentially contributing to miscarriage (Tang et al., 2024). The release of DAMPs during ferroptosis can lead to altered cytokine profiles, characterized by increased levels of pro-inflammatory cytokines like IL-6 and TNF-α. This cytokines shift promotes a hostile uterine environment, disrupting the balance between anti-inflammatory (Th2/Treg) and pro-inflammatory (Th1/Th17) responses, thereby increasing the risk of miscarriage (Shi et al., 2022). Ferroptosis-induced oxidative stress may impair the function of decidual natural killer cells, which are crucial for trophoblast invasion and immune tolerance. Altered decidual natural killer cells activity can lead to defective spiral artery remodeling and poor placental development, contributing to RM (Liu Z. et al., 2023).

### 5.3 Evidence of ferroptosis in RM pathophysiology

Emerging studies support the involvement of ferroptosis in RM, highlighting its potential role in pathophysiology through various lines of evidence.

Four key biomarkers; *PTPN6, GJA1, CPT1A*, and *CREB3L1*; are associated with ferroptosis and oxidative stress, serving as potential therapeutic targets for RM. Confirmed through bioinformatics analyses and clinical samples, these biomarkers are involved in critical pathways, including cytokine interaction and cellular responses to oxidative stress. Focusing on such molecules, along with promising therapeutic candidates like Epigallocatechin gallate and tofacitinib, offers encouraging strategies for targeted interventions in RM (Xie et al., 2024). Upregulation of ALKBH5 (alkB homolog 5, RNA demethylase) expression has already been identified as a critical factor in RM, with evidence showing it can rescue ferroptosis in cytotrophoblasts by enhanced expression of *ferritin light chain (FTL)*. Thus, this suggests a potential epigenetic regulatory mechanism that might contribute to the pathogenesis of RM (Qin et al., 2024). Ferroptosis has also been implicated in RM pathogenesis through environmental toxicants. Benzo[a]pyrene (BaP) and its metabolite benzo[a]pyrene-7,8-dihydrodiol-9,10-epoxide (BPDE) have been shown to induce ferroptosis in endothelial cells, impair angiogenesis, and contribute to pregnancy loss. These observations underscore the toxicological impact of environmental pollutants on reproductive health and pregnancy outcomes (Zhang et al., 2023).

Animal models have been instrumental in elucidating the role of ferroptosis in RM. For instance, studies using these models have demonstrated that ferroptosis can be induced by oxidative stress and hypoxia, leading to trophoblast dysfunction and miscarriage (Aslanian-Kalkhoran et al., 2022). The CBA/J*DBA/2J mouse model is often used to investigate the mechanisms associated with RM. While CBA/J mice mated with BALB/c mice served as controls, in one study these mouse groups were treated with Fer-1 (a ferroptosis inhibitor) or distilled water. The results showed that Fer-1 improved pregnancy outcomes as demonstrated by reduced rates of resorption and abortion. In addition, there was a significant reduction in iron overload and MDA levels, along with increased levels of GSH, GPX, and Solute Carrier Family 7 Member 11 (SLC7A11) (markers of ferroptosis) after treatment with Fer-1. This reduces the expression of *ACSL4* (which induces ferroptosis) (Lai et al., 2024; Ansariniya et al., 2022). These models provide valuable insights into the mechanisms of RM and potential therapeutic targets.

Histopathological studies of placental tissues from RM patients have revealed increased expression of ferroptosis-related genes, such as *FTL*, regulated by ALKBH5. This suggests that ferroptosis contributes to placental dysfunction in RM (Qin et al., 2024). The presence of oxidative stress markers and ferroptosis-related damage in placental tissues further supports the involvement of ferroptosis in RM pathophysiology (Xie et al., 2024).

## 6 Potential therapeutic approaches

RM is a multifaceted condition in reproductive medicine requiring specialized diagnostic and therapeutic strategies. Despite identifying potential causes and implementing targeted interventions, treatment outcomes vary significantly. A comprehensive diagnostic evaluation includes a detailed medical history, physical examination, and screening for antiphospholipid syndrome and uterine anomalies (Giouleka et al., 2023). Immunological factors, such as human leukocyte antigen (HLA) incompatibilities and ABO blood group discrepancies, are also relevant. Targeted immunological treatments have shown promise in improving clinical outcomes (Aliyeva et al., 2024).

A variety of strategies are integrated within the therapeutic frameworks addressing RM.

L-thyroxine administration has demonstrated efficacy in over 50% of cases involving thyroid peroxidase antibody syndrome (Mohammad, 2023), Ongoing randomized controlled trials are assessing hydroxychloroquine’s potential to enhance live birth rates in idiopathic RM (Talbot et al., 2024). The administration of IVIG therapy for RM may confer certain advantages, particularly in cases of secondary RM or among women possessing a documented history of four or more miscarriages. Contemporary research has indicated that the early application of high-dose IVIG may potentially diminish miscarriage rates, particularly in instances attributed to chromosomal anomalies. Nevertheless, the effectiveness of this treatment remains inadequately validated; consequently, it necessitates further investigation employing standardized protocols to elucidate the role of IVIG in the therapeutic management of RM (Ma et al., 2023). Lymphocyte Immunization Therapy (LIT) holds promise in improving pregnancy outcomes and increasing live birth rates in those suffering from unexplained RM. By modulating immune responses characterized by reduced levels of pro-inflammatory markers (TNF-α, IFN-γ) combined with increased Th2 and Treg cell ratios and TGF-β1 concentrations. LIT represents a potentially encouraging therapeutic approach in dealing with immunological facets of unexplained RM, though more large-scale randomized trials are needed to establish its efficacy and safety fully (Palanivel et al., 2023).

In addition, preimplantation genetic testing for aneuploidy is often performed, though it remains controversial whether this decreases the risk of RM (Table 2; Yan, 2023).

**TABLE 2:** Advances in the therapeutic management of recurrent pregnancy loss: current strategies.

Method	Description	References
Medical history	Includes detailed health history, physical examination, and screening for conditions such as antiphospholipid syndrome, uterine anomalies, and immunological factors like HLA incompatibilities.	Giouleka et al. (2023), Aliyeva et al. (2024)
L-Thyroxine Administration	Used in cases with thyroid peroxidase antibody syndrome.	Mohammad (2023)
Hydroxychloroquine	Investigated for idiopathic RM.	Talbot et al. (2024)
PGT	Used to screen for aneuploidy.	Yan (2023)
IVIG	May reduce miscarriage rates in secondary RM or women with a history of four or more miscarriages.	Ma et al. (2023)
LIT	Modulates immune responses in unexplained RM by reducing TNF-α, IFN-γ and increasing Th2/Treg cells and TGF-β1 levels.	Palanivel et al. (2023)

RM: recurrent miscarriage, PGT: preimplantation genetic testing, IVIG: intravenous immunoglobulin, LIT: lymphocyte immunotherapy, HLA: human leukocyte antigen, TNF-α: tumor necrosis factor alpha, IFN-γ: interferon gamma, Th2: T helper type 2, Treg: Regulatory T cells, TGF-β1: Transforming Growth Factor Beta 1.

Ferroptosis has been implicated in poor embryo implantation and decidual tissue damage in RM. Alpha-lipoic acid (ALA) mitigates ferroptosis by activating the PPARγ/NRF2/GPX4 pathway, enhancing antioxidant defenses, and improving pregnancy outcomes in unexplained RM. This study demonstrates that by lowering ferroptosis and oxidative stress, ALA enhances pregnancy outcomes in patients with unexplained RM (Zhao et al., 2024). Therapeutic interventions targeting the mechanisms of ferroptosis to prevent RM might also include enhancing GPX4 activity, replenishing GSH levels to reduce lipid peroxidation in decidual stromal cells, utilizing ferroptosis inhibitors such as liproxstatin-1, and modulating ferroportin expression to decrease iron accumulation and promote a successful pregnancy outcome (Sun et al., 2023).

*CDGSH Iron Sulfur Domain 2* (*CISD2*) has been identified as a crucial gene in RM, serving as a significant therapeutic target associated with ferroptosis. The downregulation of *CISD2* facilitates the induction of ferroptosis in HTR-8/SVneo and human primary extravillous trophoblast cells. A marked reduction in the expression levels of ferroptosis-related regulatory proteins, particularly GPX4 and FTH1, was distinctly observed (Shangguan et al., 2024). The lnc-HZ06/HIF1α-SUMO/NCOA4 axis emerges as a crucial mechanism linking hypoxia-induced ferroptosis in trophoblast cells to recurrent pregnancy loss. Suppression of lnc-HZ06 or NCOA4 significantly reduced ferroptosis and alleviated miscarriage in hypoxia-exposed animal models, underscoring potential therapeutic strategies for addressing unexplained RM by targeting ferroptosis-driven cellular dysfunction (Tian et al., 2024). Decreased expression of the long non-coding RNA H19 in spontaneous abortion was associated with the upregulation of Bax levels and downregulation of both Bcl-2 and GPX4 expressions. These findings suggest that H19 participates in spontaneous abortion by modulating apoptosis and ferroptosis pathways, which may provide insights for developing therapeutic strategies (Bai and Tang, 2021).

The implications of the study have identified ferroptosis as a viable pathway for therapeutic intervention in the context of RM, substantiating the assertion that the modified Shoutai Pill effectively mitigates this particular mode of cellular demise. The Jianwei Shoutai Pill demonstrated an ability to attenuate ferroptosis both *in vivo* and *in vitro* through mechanisms that involve reducing iron accumulation, lipid peroxidation, and reactive oxygen species, while simultaneously enhancing antioxidant defenses via increased expression of GPX4 and SLC7A11. These results suggest that Jianwei Shoutai Pill represents a promising therapeutic agent for the treatment of RM by modulating mechanisms associated with ferroptosis (Lai et al., 2024). It posits that Insulin-Like Growth Factor 2 mRNA Binding Protein 3 (IGF2BP3) may act as a pivotal regulator involved in RM through its role in modulating trophoblast cell migration, apoptosis, and ferroptosis. The reduced expression of IGF2BP3 in the villous tissues of RM patients correlated with a decrease in mRNA stability of GPX4, thereby promoting ferroptosis and hindering trophoblast invasion. These findings suggest that manipulating IGF2BP3 could lead to novel therapeutic approaches for RM by influencing the associated ferroptosis-related pathways (Dai et al., 2024). The investigation highlights ferroptosis as a potentially significant therapeutic target for unexplained recurrent spontaneous abortion, emphasizing the regulatory function of *circadian rhythm gene cryptochrome 2 (CRY2)* associated genes. The upregulation of *CRY2* was found to facilitate ferroptosis in trophoblast cells, thereby disrupting epithelial-mesenchymal transition and reducing migratory capacity, while Brain and muscle arnt-like (BMAL1) acted as a modulating counter-regulator. Importantly, the application of ferrostatin-1 successfully mitigated the ferroptosis and EMT-related dysfunctions, indicating promising pathways for therapeutic intervention (Li et al., 2024). Ferroptosis-related genes may serve as potential therapeutic targets for addressing early missed abortion, a significant pathogenic occurrence in the initial phase of pregnancy. Important discoveries highlight the functions of important genes identified by gene ontology and pathway enrichment studies linked to p53 signaling, mitophagy, and protein degradation pathways, such as *Tumor protein p53 (TP53)*, *Enhancer of Zeste Homolog 2 (EZH2*), *Tissue inhibitor matrix metalloproteinase 1 (TIMP1)*, *Solute carrier family 3 member 2 (SLC3A2),* and *Gamma-aminobutyric acid receptor-associated protein-like 2 (GABARAPL2)* (Zeng et al., 2025). These findings make the development of biomarker-based approaches to enhance the early detection and management of recurrent pregnancy loss possible (Table 3).

**TABLE 3:** Emerging therapeutic approaches targeting ferroptosis pathways for the management of recurrent pregnancy loss.

Method	Mechanism/Description	References
ALA	Prevents ferroptosis by activating the PPARγ/NRF2/GPX4 pathway, reducing oxidative damage and lipid peroxidation, thereby improving pregnancy outcomes in unexplained RM	Zhao et al. (2024)
Ferroptosis Inhibitors	Liproxstatin-1 and ferrostatin-1 inhibit lipid peroxidation and oxidative damage in decidual and trophoblast cells.	Sun et al. (2023), Li et al. (2024)
GPX4 and GSH Modulation	Enhances GPX4 activity and replenishes GSH levels to reduce ferroptosis in decidual stromal cells.	Sun et al. (2023)
CISD2 Targeting	Downregulation of CISD2 induces ferroptosis in trophoblast cells.	Shangguan et al. (2024)
Jianwei Shoutai Pill	Reduces iron accumulation, lipid peroxidation, and ROS, while increasing GPX4 and SLC7A11 expression to inhibit ferroptosis *in vivo* and *in vitro*.	Lai et al. (2024)
IGF2BP3 Regulation	Enhances mRNA stability of GPX4, mitigating ferroptosis and promoting trophoblast cell invasion to improve RM outcomes.	Dai et al. (2024)
Circadian Rhythm Modulation	Upregulation of Circadian Rhythm Modulation induces ferroptosis, while BMAL1 acts as a counter-regulator; ferrostatin-1 prevents ferroptosis and restores EMT and migratory capacity.	Li et al. (2024)
lnc-HZ06/HIF1α-SUMO/NCOA4 Axis	Suppression of lnc-HZ06 and NCOA4 mitigates hypoxia-induced ferroptosis, reducing miscarriage in animal models.	Tian et al. (2024)
lncRNA H19 Modulation	Decreased H19 expression is linked to upregulated Bax and downregulated Bcl-2 and GPX4, highlighting its role in apoptosis and ferroptosis pathways in RM.	Bai and Tang (2021)
Ferroptosis-related genes	Identifies genes (e.g., *TP53*, *EZH2*, *TIMP1*, *SLC3A2*, *GABARAPL2*) associated with ferroptosis, offering biomarker-based approaches for early detection and intervention in missed abortion and RM.	Zeng et al. (2025)

ALA: Alpha-Linolenic Acid,GPX4: Glutathione Peroxidase 4, GSH: glutathione, CISD2: CDGSH, Iron Sulfur Domain 2, SLC7A11: Solute Carrier Family 7 Member 11, IGF2BP3: Insulin-Like Growth Factor 2 mRNA, Binding Protein 3, RM: Recurrent miscarriage, EMT: Epithelial-Mesenchymal Transition, HIF1α: Hypoxia-Inducible Factor 1 Alpha, lnc-HZ06: Long Non-coding RNA HZ06, NCOA4: Nuclear Receptor Coactivator 4, lncRNA: Long Non-coding RNA, ROS: reactive oxygen species, TP53: Tumor Protein 53, EZH2: Enhancer of Zeste Homolog 2, TIMP1: Tissue inhibitor matrix metalloproteinase 1, SLC3A2: Solute Carrier Family 3 Member 2, GABARAPL2: Gamma-aminobutyric acid receptor-associated protein-like 2.

## 7 Conclusion

Ferroptosis, defined as a distinctive form of iron-dependent cell death featuring lipid peroxidation and oxidative stress, has come to be recognized as an essential pathophysiological process in RM. Evidence has shown that the interplay between ferroptosis and trophoblast dysfunction, dysregulation of the immune system, and placental oxidative damage disrupts the delicate balance crucial for the success of a pregnancy. Abnormal iron metabolism, oxidative stress, and pathways related to ferroptosis have been well-marked to promote cellular damage in RM.

The therapeutic avenues related to modulating ferroptosis are promising yet complicated. Interventions targeting support for antioxidant defenses, iron homeostasis modulation, or prevention of lipid peroxidation have been shown to attenuate trophoblast dysfunctions caused by ferroptosis. Treatment modalities involving the administration of ferroptosis inhibitors, GPX4 activators, and classical and modern interventions, such as Jianwei Shoutai Pill and ferrostatin-1, now offer new avenues to modulate. Moreover, elucidations of epigenetic and gene regulatory mechanisms, such as lnc-HZ06, NCOA4, and IGF2BP3, provide much more profound insights into the ferroptosis-driven molecular pathways and their potential effects on RM.

Future studies should translate these findings into clinical practice, with priority given to the development of biomarkers for early diagnosis and precision-targeted treatment modalities. The integration of ferroptosis-targeted approaches within the comprehensive therapeutic paradigms has the potential to revolutionize the management of RM by addressing this complex condition with a mechanistic and tailored approach. Such developments hold the potential to alleviate the great physical, psychological, and financial burdens faced by affected individuals and their families.
